# Sex and gender differences in community-acquired pneumonia

**DOI:** 10.1007/s11739-022-02999-7

**Published:** 2022-07-19

**Authors:** Bernadette Corica, Francesco Tartaglia, Tania D’Amico, Giulio Francesco Romiti, Roberto Cangemi

**Affiliations:** Department of Translational and Precision Medicine, Sapienza-University of Rome, Viale del Policlinico 155, 00162 Rome, Italy

**Keywords:** Pneumonia, Sex, Gender, COVID-19, Review

## Abstract

Awareness of the influence of sex ands gender on the natural history of several diseases is increasing. Community-acquired pneumonia (CAP) is the most common acute respiratory disease, and it is associated with both morbidity and mortality across all age groups. Although a role for sex- and gender-based differences in the development and associated complications of CAP has been postulated, there is currently high uncertainty on the actual contribution of these factors in the epidemiology and clinical course of CAP. More evidence has been produced on the topic during the last decades, and sex- and gender-based differences have also been extensively studied in COVID-19 patients since the beginning of the SARS-CoV-2 pandemic. This review aims to provide an extensive outlook of the role of sex and gender in the epidemiology, pathogenesis, treatment, and outcomes of patients with CAP, and on the future research scenarios, with also a specific focus on COVID-19.

## Introductions

Sex and gender represent two key aspects to better understand the epidemiology and mechanistic pathways of different diseases in almost all medical fields. Although often incorrectly used as synonyms, “sex” and “gender” terms are not interchangeable. Sex refers to a biological classification that distinguishes males from females based on chromosomal, hormonal, and anatomical differences. Gender, instead, refers to cultural attitudes, roles, and behaviors stereotypically associated with sex, which shape self-identity [1]. While sex may influence immune response directly, gender may influence habits that determine exposure to microorganisms and healthcare-seeking behaviors (see Table 1 for details in Sex and Gender differences).

**Table 1:** Sex and Gender-related characteristics [118]

Sex	Gender
Male and female	Masculine and feminine
Defined by karyotype	Multifaced and complex
Not modifiable	Culturally defined
Anatomy differences	Can change over time
Endocrine hormones differences (e.g., Testosterone VS Estrogens)	Roles and responsibilities differences (e.g., construction, defense VS caring, service jobs)
Gene expression differences	Entitlements differences (e.g., higher workforce participation, financial autonomy VS Inferior healthcare to men, financial dependence)
	Different attributes differences (e.g., risk-taking, aggression VS fragile, emotional)

The role of both sex and gender has poorly been explored in the pathophysiology of infectious disease, and little is known about their contribution in the epidemiology, clinical course, treatment response, and ultimately outcomes of community-acquired pneumonia (CAP).

Globally, pneumonia represents the fourth leading cause of death worldwide and the most lethal communicable disease [2]. The 2019 Global Burden of Diseases (GBD) study showed that Lower Respiratory Tract Infections (LRTIs) were responsible for > 2.49 million deaths (> 1.29 million in men and almost 1.2 million in women), with highest mortality rates at extreme ages (1.23 million deaths among > 70 year old patients, and 672.000 deaths among < 5 year old patients). However, both morbidity and mortality showed a decreasing trend over the last decades [2, 3]. In Europe, CAP is responsible for at least 23.000 deaths/year [4].

Epidemiology of CAP can vary consistently between world regions, because of differences in risk factor distribution, healthcare systems development and accessibility, prevention policies, climate, and other factors [5]. As recently shown, beyond the mortality and morbidity burden, CAP induces several cardiovascular complications [6]; moreover, different aspects of CAP could be influenced by sex and gender, like the smoking habit, which is higher in men and is associated with a higher incidence of CAP [7], or the differential immune response to the infections, which could have an important role in determining different outcomes among men and women [8]. In this review, we highlight the most recent evidence on the differences in CAP in the light of sex and gender determinants.

## Epidemiology and etiology of CAP: does sex and gender matter?

According to the 2019 GBD study, LRTI was responsible for 489 million incident cases (257 million in men, 232 million in women) and 11 million prevalent cases (5,8 million in men and 5,2 million in women) globally in 2019, with an incidence that is slowly but steadily increasing since 1990 [3, 9]. A previous GBD study focused on LRTI shows that the most affected populations are children of < 5 years of age (107.7 episodes per 1,000) and adults of > 70 years of age (155.4 episodes per 1000) [10], confirming the well-known *U*-shaped incidence of pneumonia in terms of age.

In Europe, the overall annual incidence of CAP in adults was found between 1.07 and 1.7/1000 person-years [11]. Incidence raises to 14 cases per 1,000 person-years among those of ≥ 65 years of age, and men were more affected than women, with a highest incidence of 23.1/1000 person-years found in men with Chronic Obstructive Pulmonary Disease (COPD) [11]. However, the epidemiology of CAP is largely influenced by geographical differences; furthermore, pandemic outbreaks such as those caused by influenza virus H1N1 in 2009 and, more recently, SARS-CoV-2, are responsible for significant temporal variations in the incidence of LRTI worldwide.

Sex differences in CAP incidence have been reported in several epidemiological studies, although there are still few sex-disaggregated data available. Table 2 summarizes recent evidence on sex-based differences in epidemiology and prognosis of CAP. In all the studies, the incidence of CAP was higher in males and increased with age in both sexes. Sex difference was confirmed in age-stratified analysis [12], but was mostly present in the elderly (≥ 65 years) [13, 14].

**Table 2:** Sex-disaggregated data on CAP incidence and severity

Study	Study type and years of data	Country	Number of patients	Incidence rates by sex	Outcomes by sex
Restrepo et al. [119]	Retrospective, 1999–2001	USA	730 patients with CAP	Not reported	Patients were more likely males in ICU (88%) than ward (75%) (*p* = 0.001)
Reade et al. [54]	Prospective, 2001–2003	USA	2.183 patients with CAP	Not reported	Men had a higher ICU admission rate (4.4% vs 2.2%, *p* = 0.001) and a higher risk of death at 30-days (7% vs. 4.5%, *p* = 0.01), 90-days (11.4% vs. 8.6%, *p* = 0.02) and one year (21% vs. 16%, *p* = 0.002) Adjusted HR for 1-year mortality = 1.29, (95% CI = 1.05–1.59; *p* = 0.04)
Millet et al. [12]	Retrospective, 1997–2011	United Kingdom	65.000 CAP cases from more than 1,5 million patients, all ≥ 65 years old	M: F = 56: 44. Incidence rate: 8.60 (M) vs 7.53 (F) /1000 person-years; after standardizing for age: 8.31–11.09 (M) vs 5.56–7.65 (F) /1000 person-years	Not reported
Arnold et al. [120]	Secondary analysis of clinical trial, 2001–2011	17 countries	6.718 patients with CAP	M: F = 60: 40	Adjusted HR for time to clinical stability = 0.91 (95% CI 0.85–0.97; *p* = 0.05) Adjusted HR for length of stay = 0.94 (95% CI 0.88–1.01; *p* = 0.089) Adjusted RR for in-hospital mortality = 1.04 (95% CI 0.86–1.24; *p* = 0.717) Adjusted RR for 28-day mortality = 1.15 (95% CI 1.02–1.30; *p* = 0.018)
Kolditz et al. [121]	Prospective, 2007–2013	Germany	3.427 patients with CAP	Not reported	No sex-related difference in developing of “emergency CAP”
Rivero-Calle et al. [13]	Retrospective, 2009–2013	Spain	28.400 CAP cases from 2,3 million patients	Incidence rate: 5.04 (M) vs 4.26 (F) /1000 person-years. In 18–65 years old: 2.18–5.75 (M) vs 1.47–5.21 (F) /1000 person-years. In > 65 years old: 7.06–36.39 (M) vs 5.43–19.62 (F)	Not reported
Gonzalez Quero et al. [122]	Prospective, 2012–2016	Spain	1389 patients with CAP	M: F = 64: 36	IHM = 3% in both groups
Corrado et al. [123]	Retrospective, 2010–2014	USA	154.000 patients with CAP	Mean annual age-adjusted hospitalization rates for CAP: 552.6 (M) vs 429.2 (F) /100.000 population/year. RR for male sex = 1.3 (95% CI 1.3–1.3)	Not reported
de Miguel-Diez et al. [86]	Retrospective, 2004–2013	Spain	960.000 admissions for CAP	M: F = 61: 39. Incidence was higher in males in all age groups	Female sex is a risk factor for IHM: OR = 1.05 (95% CI 1.04–1.06)
Alsawas et al. [82]	Retrospective, 1995–2015	USA	13.000 cases of pneumonia	M: F = 55: 45	Men had a higher 30-days mortality: adjusted OR = 1.19 (95% CI = 1.06–1.34)
Fassmer et al. [124]	Retrospectiv, 2010–2014	Germany	19.200 cases of pneumonia from 127.000 nursing homes’ patients, all ≥ 65 years old	Incidence rate: 20.9 (M) vs 9.6 (F) /100 person-years. HR for male sex = 1.88 (1.83–1.94)	Not reported
Pessoa et al. [83]	Retrospectiv, 2000–2014	Portugal	549.000 hospitalizations due to CAP	M: F = 55: 45	Male sex is a risk factor for IHM: adjusted OR = 1.261 (95%CI 1.243–1.280)
Peyrani et al. [87]	Secondary data analysis, 2014–2016	USA	7.449 patients with CAP	Not reported	No sex-related difference was found in groups with clinical improvement, clinical failure or non-resolving pneumonia
Sun et al. [14]	Retrospectiv, 2016	China	1.48 million CAP episodes from 427 million patients	Incidence rate: 7.32 (M) vs 6.93 (F) /1000 person-years; after standardizing for age: 9.52 (M) vs 8.54 (F) /1000 person-years	Not reported
de Miguel-Yanes et al. [16]	Retrospectiv, 2016–2019	Spain	519.000 patients with CAP	M: F = 59: 41. Incidence rate: 429.59 (M) vs 283.3 (F) /100.000 inhabitants. Incidence rate ratio (IRR) = 1.47 (95% CI 1.45–1.50)	IHM: 12.5% (M) vs. 12.2% (F) before PSM, and 12.9% (M) vs. 12.2% (F) after PSM (= a 5.7% higher relative risk among men). Male sex was a risk factor for IHM: OR = 1.13 (95% CI 1.10–1.15)

Studies are ordered according to publication year. Where not otherwise specified, patients’ number indicates the total studied population, not the number of CAP cases

*CAP* community-acquired pneumonia, *ICU* intensive care unit, *HR* hazard ratio, *OR* odds ratio, *RR* risk ratio, *IHM* in-hospital mortality, *PSM* propensity score matching, 95% CI 95% confidence interval

Moreover, sex-based differences were observed also for the prevalence of several comorbidities: while both sexes were frequently affected by metabolic disease (27.8% of males, 26.9% of females) and cardiovascular disease (20.5% of males and 15% of females), diabetes and smoking were mainly found in men (17.9% and 15.5%, respectively), while depression and anemia were more frequently found in women (20.0% and 15.8%, respectively) [13], although another study has found anemia to be more commonly reported in males [15]. A large Spanish cohort confirmed that men with CAP presented with overall more comorbidities, although with lower prevalence of heart failure (25.5% in women, 20.5% in men), dementia (11.3% vs 7.3%), and rheumatoid disease (3.7% vs 1.8%) [16]. Finally, type-2 diabetes mellitus (T2DM) [17] and COPD [18] were associated with a higher CAP incidence in both sexes, but disproportionally more in men than women. Moreover, males reported a higher rate of comorbidities and at-risk habits [19] and this likely plays a significant role in determining their higher incidence of CAP. Overall, it is also possible that sex differences reported in these cohorts were influenced by the patients' age and geographical locations of the studies.

Beyond sex, data regarding gender-related differences in the epidemiology of CAP are scarce and underline how these aspects are poorly studied in this clinical scenario. First, gender differences entail job segregation, meaning that some jobs are typically done more by men than women. Indeed, toxic exposure in the workplace has been reported higher for males than females [20, 21] and this may contribute to shaping the incidence of CAP.

The impact of gender-related aspects, however, is not negligible. A study conducted in a pediatric Bangladeshi population [22] showed that among patients hospitalized for CAP, a higher proportion of females presented with severe pneumonia as compared to males, with also a fourfold higher death rate among females. They speculated that retarded hospital presentation for female children may have a role in determining these findings, with delay in seeking medical attention when the child was of female sex. [22] They also underline how similar results for other diseases were found in other studies. From a general point of view, these findings reinforce the importance of cultural and gender-specific variables in the epidemiology of CAP, as well as other diseases. Unsurprisingly, delayed hospital presentation in females has been reported for other acute medical conditions [23], and this may also contribute to the lower CAP incidence observed in women, with a trend toward seeking hospital assistance only when clinical conditions get worse, whereas mild cases—which will likely resolve spontaneously—do not arrive at the attention of the Emergency Department and therefore are under-reported in epidemiological reports.

## Sex differences in pathophysiology of CAP

**Fig. 1:**
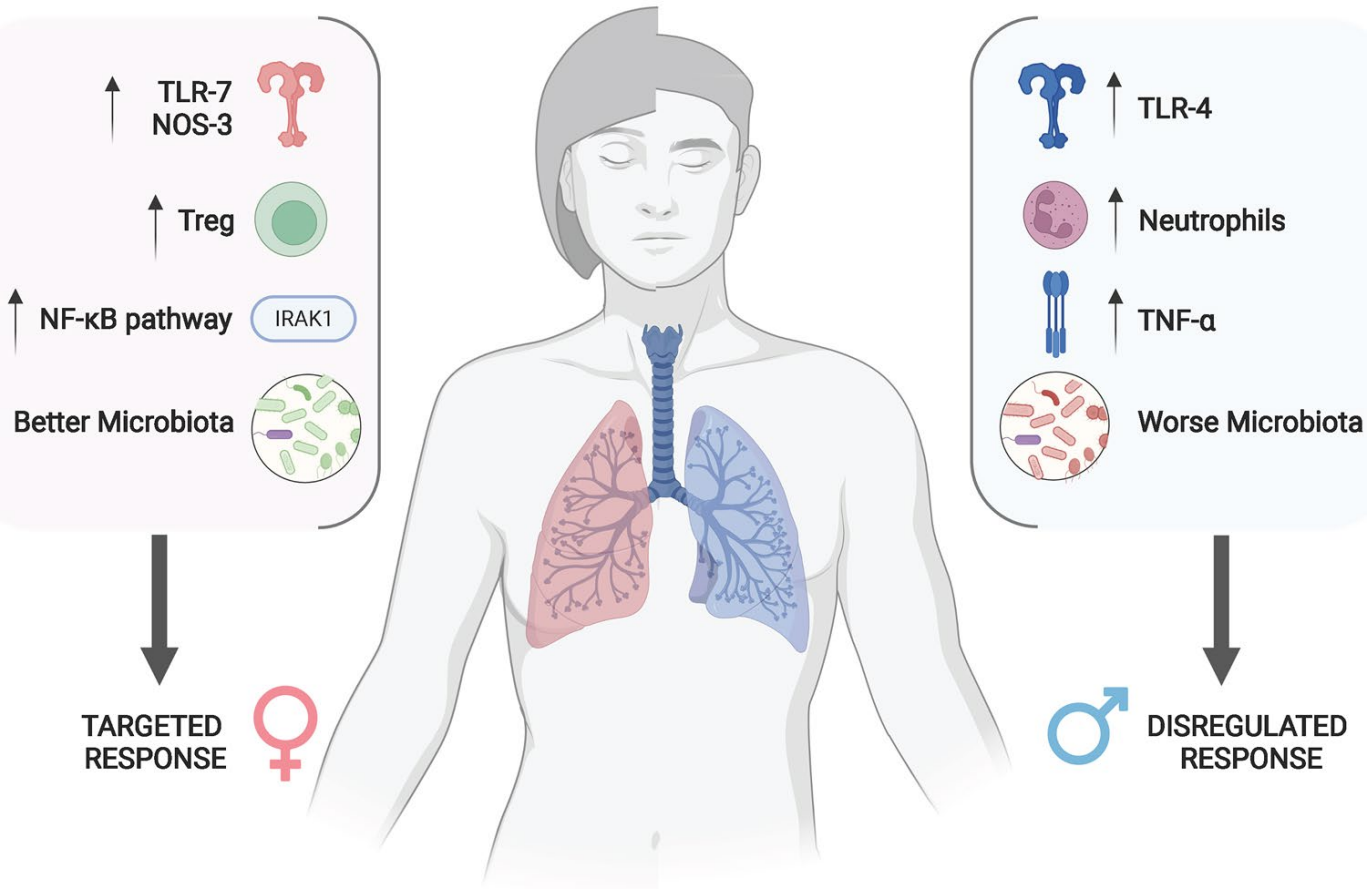
Factors determining a different response to pneumonia in men and women (created with Biorender.com). *IRAK-1* IL-1 receptor-associated kinase-1, *NOS-3* nitric oxide synthase-3, *TLR-7* toll-like receptor-7, *TLR-4* toll-like receptor-4, *Treg* T regulatory lymphocytes, *TNF-α* tumor necrosis factor-αs

Traditionally, CAP is believed to be caused by the translocation of a virulent microorganism from the oro- and nasopharynx to the lower respiratory tract [5]. Many host-related factors, such as preceding viral infection, smoke exposure [7], and COPD, facilitate the transition from colonization to infection [24]. Furthermore, pathogens’ virulence and the host immune response create damage to the lung parenchyma. Figure 1 presents a summary of sex-related differences in CAP pathophysiology.

Defenses against pneumonia include anatomical barriers (mucus, mucociliary clearance, and intercellular junctions), as well as innate and adaptive immunity. Excessive inflammation is controlled through tissue resilience mechanisms, activated by anti-inflammatory cytokines like IL-10 [5, 8].

On the other hand, local factors may influence the pathophysiology of CAP. The lower respiratory tract host different taxa [25] and their individual composition may influence the immune response [26]. A similar pattern has been observed for the gut microbiota, which also influences immune responses in the lung [27]. Sex-related differences have been found in gut microbiota’s composition [28], its influence on the immunity system [29], and its interaction with risk factors for several diseases [30]. Therefore, it seems reasonable to infer that similar sex-related differences exist in lung microbiota as well, being perhaps one factor associated with the lower CAP incidence in women; however, this relationship has not been adequately elucidated yet.

Studies on animal models have shown several interesting findings on sex-based differences in the immune response. For example, male mice inoculated with *Streptococcus pneumoniae* showed higher levels of neutrophils, IL-17A, CXCL1, and CXCL2 than females [31], but, when mice were infected with *Pseudomonas aeruginosa*, levels of CXCL1 and TNF-α were higher in females; similarly, female mice showed higher interferon-γ levels when infected with influenza virus [32].

Moreover, macrophages from male mice expressed higher levels of Toll-Like Receptor-4 (TLR-4, a pathogen-recognition receptor used by several innate immunity cells) than females, when exposed to bacterial lipopolysaccharide (LPS) [33, 34], thus developing an increased inflammatory response. However, TLR4 expression in macrophages from mice was found to be reduced by androgens [34] and increased by estrogens [35], increasing the complexity in understanding the relationship between sex, sexual hormones, and immune response. Like TLR4, also TLR7 shows differences among sexes. Its expression is higher in female mice [36], thus causing a higher production of IFN-α in females than males; this was confirmed in studies on humans [37]. The reason for this diversity may be found in the X chromosome, which hosts several genes involved in innate immunity. Female cells are characterized by the casual inactivation of one X chromosome, leading to X-linked mosaicism in women. This entails the presence of polymorphisms that give women a potential expanded immune repertoire when compared to men [38, 39]. Moreover, some genes escape the X inactivation process and are therefore expressed twice as much as the others, which is likely the case of TLR7 [39]. Similarly, IL-1 receptor-associated kinase-1 (IRAK-1) gene may also escape X chromosome inactivation, leading to a higher NF-κB pathway activation in females [39], and therefore a decreased susceptibility to infections; this was found in both mice and humans [39]. Finally, mouse models also demonstrated that surfactant–protein-A likely plays a role in innate lung response by the clearance of pathogens by alveolar macrophages [40]. It has been demonstrated that differences in innate response may rely on the different surfactant–protein-A variants among sexes [41]. Another factor contributing to female resistance to pneumonia, elegantly shown by Yang et al. [42], may be the higher activation of nitric oxide synthases (NOS)-3, which produces bacteria-killing factors called reactive nitrogen intermediates in macrophages both in mice and in humans. NOS-3 levels are increased by estrogens and statins, and authors found that receiving estrogenic therapy, statin therapy, or both was associated with a reduced incidence of pneumonia requiring hospitalization in women. [42] While this may represent an interesting therapeutic target for future developments, it only explains partially the therapeutic effects of estrogens in pneumonia. In fact, by binding to specific response elements hosted in the promoter of several genes [43], estrogens produce a broad pro-inflammatory effect: they enhance neutrophils recruitment in lungs in female mice infected with influenza A virus [44] and increase TLR4 expression on macrophages. Moreover, they also promote the resolution of inflammation after pneumonia, through the action of T regulatory lymphocytes [45]. On the other hand, other sex hormones (such as androgens and progesterone) may exert anti-inflammatory effects [8], for example by antagonizing NF-kB pathways [46].

In studies on humans, men showed a higher inflammatory response in airways, consisting mainly of neutrophils and cytokines such as IL-8, IL-1β, and TNF-α [39] which is likely responsible for higher incidence and worst outcomes. In vitro analysis of human peripheral cells highlighted that, like macrophages in mice, human male neutrophiles expressed higher levels of TLR4 when exposed to LPS than female ones [47] and released more TNF-α.

Furthermore, the prevalence of smoking habits is different among the sexes [48]. Pro-inflammatory effect has been previously demonstrated, by inducing the production of TNF-α, IL-1, IL-6, and IL-8 with consecutive recruitment of neutrophils and macrophages, and consequently damage to the lung tissue. Moreover, cigarette smoke may also influence adaptive immunity by changing T-cell subtypes’ prevalence [49] and B-cell deposition in blood and tissues and decrease immunoglobulin production [50].

Finally, data on pregnancy are particularly limited. Pregnancy imposes a condition of relative immunodepression, which may lead to worse outcomes for both mother and child [51], although the risk of developing pneumonia does not seem higher when compared to nonpregnant women [52]. Further studies are needed to elucidate better how pregnancy can influence the risk of CAP and associated outcomes.

Despite this amount of evidence, the overall understanding of sex differences in immune response in CAP remains limited. A hypothetical comprehensive model may show that males’ response to pneumonia is easily dysregulated, while female one is more targeted and hence less destructive, but further research is needed to confirm these hypotheses and to test therapeutic implications.

## Diagnosis, management, and prevention

CAP is usually clinically suspected when cough, fever, expectoration, and dyspnea are presented acutely, along with suggestive radiological findings. However, the clinical presentation can vary significantly, and there is no definitive evidence on sex differences in symptomatology. Some authors speculated that the delay on antibiotic treatment reported in women with complicated CAP may be attributable to their milder clinical presentation [53], and others confirmed the greater severity in men [54]. This suggests that there is a difference in the symptomatology at the onset. Beyond that, it is clear that symptoms may be less evident in anergic patients (e.g., immunocompromised, elderly patients) [55].

Chest radiograph (CXR) is fundamental for the diagnosis of CAP. However, other imaging techniques with good sensitivity and specificity are currently employed to diagnose CAP, including lung ultrasound, which has the advantage to be X-ray free and suitable for critically-ill patients at the bedside, and also useful in pregnant women [56].

Microbiological diagnosis (e.g., naso-pharyngeal swabs, blood samples, and good-quality sputum) is recommended in patients requiring hospitalization, but it cannot be obtained in up to half cases of CAP [57]. Sex-related differences are evident also in the microorganisms responsible for CAP and isolated through these techniques [16]. *Streptococcus pneumoniae*, Influenza virus, and other viruses were found in women more than men, while *Candida*, *Aspergillus*, *Escherichia coli*, *Pseudomonas aeruginosa*, *Legionella pneumoniae*, and *Klebsiella pneumoniae* were reported more frequently in men [19, 58, 59]. The difference in microorganisms isolated in CAP may reflect sex imbalance in comorbidities or a different exposure to pathogens due to gender roles: for example, men may be more exposed to Legionella in the workplace, especially in developing countries [58]. However, there is still limited evidence regarding sex-based differences in the microbiology of CAP.

Management of CAP must include comorbidities and the risk of systemic complications that could require hospitalization. Several factors can contribute to worsening outcomes; most of them are included in the pneumonia severity risk scores that are frequently used to stratify patients in a class of risk. Risk prediction is needed to identify patients who need hospitalization and higher intensity of care, including the need for admission to Intensive-Care-Unit (ICU). Although sex and gender are believed to influence the prognosis of patients with infectious diseases, not all risk assessment models for CAP take sex into account. An overview of the most common scores used in clinical practice is reported in Table 3; the most used are Pneumonia Severity Index (PSI) [60] and CURB-65 [61]. The PSI is a score that predicts morbidity and mortality. Sex is among the variables included, with women that are attributed ten points less than males regardless of age and other comorbidities; since the score gives one point each year the risk of a woman is comparable to a 10-year younger man, this is consistent with a predicted lower risk of outcomes in females.

**Table 3:** Variables included in each pneumonia score and their performances in predicting ICU admissions

Score	Variables																				Risk classes
	Mechanical Ventilation	Shock	Age	Sex	Comorbidities	Confusion	Heart Rate	Blood Pressure	Respiratory Rate	Temperature	pO2/FiO2	Arterial pH	Multilobular infiltrate	Hematocrit	Sodium	Glycemia	Urea or Urine Output	Albumin	Leucocytes	Thrombocytes	
PSI			x	x	x	x	x	x	x	x	x	x	x	x	x	x	x				0, ≤ 70, 71–90, 91–130, ≥ 130
CURB-65			x			x		x	x								x				≤ 1, 2, ≥ 3
CRB-65			x			x		x	x												
CURB						x		x	x								x				Severe if ≥ 2 variables
CORB						x		x	x		x										
ATS 1993	x	x						x	x		x		x				x				Severe if ≥ 1 variable
ATS 2001	**M**	**M**						**m**			**m**		**m**								Severe if 1 M or ≥ 2 m
ATS/IDSA 2007	**M**	**M**				**m**		**m**	**m**	**m**	**m**		**m**				**m**		**m**	**m**	Severe if 1 M or ≥ 3 m
SMART-COP						x	x	x	x		x	x	x					x			0–2, 3–4, 5–6, ≥ 7
SCAP			x			x		x	x		x	x	x				x				0, 1–9, 10–19, 20–29, ≥ 30
REA-ICU			x	x	x		x		x		x	x	x		x		x		x		≤ 3, 4–6, 7–8, ≥ 9

*ATS/IDSA* American thoracic society/infectious diseases society of America, *CRB-65* confusion, respiratory rate ≥ 30, blood pressure < 90 mmHg (systolic) or ≤ 60 mmHg (diastolic), Age ≥ 65 years, *CURB-65* Confusion, BUN > 7 mmol/L, Respiratory rate ≥ 30, Blood pressure < 90 mmHg (systolic) or ≤ 60 mmHg (diastolic), Age ≥ 65 years, *M* major criteria, *m* minor criteria, *PSI* pneumonia severity index, *REA-ICU* risk of early admission to intensive care unit, *SCAP* severe community-acquired pneumonia score, *SMART-COP* systolic blood pressure, multilobar infiltrate, albumin, respiratory rate, tachycardia, confusion, low oxygen, low pH

The CURB-65 [61] is a more recent mortality-predicting score. This score is easier to use in a clinical setting, but, when compared to PSI, has a lower discriminative power [62]. Its use is recommended by the British Thorax Society guidelines [63], whereas the American Thoracic Society (ATS) suggest the use of PSI [57]. To note, PSI, CURB-65 and CRB-65 (a reduced model of CURB-65), were designed to predict 30-day mortality, so they perform worse in predicting ICU admission [64] compared to other scores.

In 2007, ATS together with the Infectious Diseases Society of America (IDSA) has also developed criteria for defining severe CAP [57] (see Table 3): the presence of either one major criterion or ≥ 3 minor criteria indicate the need for ICU admission, which is predicted with a sensitivity of 83.8% and a specificity of 77.7% [65]. However, sex is not included in this score.

Several studies have proposed other scores, intending to outperform PSI [60] and CURB-65 [61] in predicting outcomes other than mortality, including SMART-COP [66], Severe Community-Acquired Pneumonia (SCAP) [67] score, and Risk of Early Admission to Intensive Care Unit (REA-ICU) score [68]. Among these three tools, only REA-ICU [68] takes sex into consideration. Beyond that, several modified versions of CURB-65 were proposed, aiming at improving its predictive performance; however, although some demonstrated higher sensitivity in predicting mortality [69], the potential contribution of sex in improving predictive ability of CURB-65 is still unknown. Although adding sex to CURB-65 may increase its discriminative ability, specific studies are needed to validate this hypothesis.

Apart from sex variables, none of the scores mentioned include gender-related variables including educational level, socio-economic status, social support, and caregiver assistance. Although the contribution of these factors was not extensively studied in patients with CAP, it is conceivable that they may play an important role in influencing the clinical course, morbidity, and mortality of CAP patients, especially outpatients. Gender-specific characteristics may also reduce medical adherence or hospital seeking for clinical worsening [70] and their role in risk stratification in CAP may be worth further investigation.

Beyond risk stratification, the timing of the initial treatment of CAP has been largely discussed. Empiric antibiotic therapy should be started within 4 h from admission [71] and anticipated at 1 h in patients with septic shock [72]. However, women are more likely to receive later antibiotic treatment, even though this does not reflect in increased mortality [53, 54]. Advanced treatments with vasopressor and non-invasive ventilation (NIV), which require other settings of care instead of general wards, depend on the complication of CAP; what is has been highlighted is that men are more likely to be admitted to ICU compared to women [54, 73]. This is probably due to the different severity of CAP at presentation, the higher risk stratification scores, and consequently the early access to ICU.

Several factors (unhealthy habits, chronic lung diseases, and medications [74]) increase the risk of CAP and prevention is fundamental, especially for those most exposed. Prevention strategies lie in changes in unhealthy habits, in particular quitting cigarette smoke, due to its role in damaging lungs’ ability to fight off infections. It is likely that prevention strategies may have differential efficacy among sexes and can be influenced by several gender-related factors, but data on these aspects—as well as the impact on outcomes—are currently scarce.

The 23-valent pneumococcal polysaccharide vaccine (PPV23), the 13-valent pneumococcal conjugate vaccine (PCV13) [75], and seasonal influenza vaccine are highly encouraged for people at high risk of pneumonia; however, response to the vaccine is not the same among males and females [76, 77] and recent data showed that women could have a more robust IgG response to the 23-valent pneumococcal polysaccharide vaccine (PPSV23), as compared to men [78]. Again, further studies are urgently needed to expand our knowledge on these issues.

On the other hand, it has been demonstrated that influenza vaccination is associated with a reduced risk of hospitalization for pneumonia as well as cardiovascular and cerebrovascular diseases, and the risk of death from all causes during seasonal influenza. This is particularly evident among the elderly. [79]

## Sex- and gender-associated outcomes

Sex and gender differences have been reported also for the clinical course and outcomes of CAP patients, especially in terms of short- and long-term mortality.

Historically, worse outcomes were reported in men with CAP, with a 30% increase in the risk of mortality in males compared to women [80]. A 2007 review [81] reports that male sex was associated with worse outcomes in terms of duration of hospitalization, more complex course of CAP, and mortality.

Table 2 summarizes recent studies that include CAP-associated outcomes according to sex. In most of them, outcomes were worse in males. Geographical differences are indeed important, as mortality was almost 20% higher in males in a US-based cohort [82], while slightly higher figures were observed in a population from Portugal [83].

Men were found to have a 13% higher in-hospital mortality (IHM) [16]. Moreover, the impact of comorbidities on IHM differed among sexes: myocardial infarction, cerebrovascular disease, and cancer had a higher impact in women [16], but further studies need to confirm this different impact on mortality, whereas T2DM [84] and COPD [85] were associated with a higher risk of IHM rate in both sexes.

Other evidence confirmed higher mortality risk in men at 30, 90, and 365 days compared to women [54]. The higher death risk in men remained significant once the Hazard Ratio (HR) was adjusted for differences in demographics, comorbidities, illness severity, and other clinical risk factors [adjusted HR 1.29, 95% Confidence Interval (CI) 1.05–1.59, *p* = 0.004], but it became non-significant when additionally adjusted for differences in baseline biomarkers concentration (TNF, IL-6, IL-10, D-dimer, antithrombin-III, and Factor IX). This may highlight the role of immunological differences on the worse outcomes compared to the higher burden of comorbidities [54].

However, these data were not confirmed in all cohorts, as reported in Table 2. A large Spanish study found a significant 5% higher risk of death in females [86], while other evidence found no difference in mortality among sexes when analyzing clinical progress [87]. Reasons for this conflicting evidence are not completely understood, but it may be due to differences in demographics and gender-specific factors, which were largely not accounted in sex-stratified analyses.

On the other hand, when considering ICU admission and advanced treatments as a surrogate of different outcomes among sexes, interesting differences were observed in some cohorts. Patients admitted to the ICU were more likely males [54, 73] and underwent more diagnostic and therapeutic interventions [bronchial fibroscopy, chest computerized tomography, dialysis, invasive mechanical ventilation (IMV), and surgery] than females, except for NIV [16].

Oppositely to sex-based differences, the effect of gender-related variables on CAP severity and outcomes has not been extensively investigated, and data on the contribution of gender are urgently needed to expand our knowledge on its impact on clinical progression and outcomes in patients with CAP.

Nevertheless, several studies have pointed out that treatment disparities may exist between men and women. As we extensively presented above, women seem to be less ill on presentation, and probably, for this reason, we can speculate that they receive later the antibiotic treatment compared to men, even when in sepsis or septic shock [53]. However, these differences may be mitigated by a higher adherence to treatment guidelines and recommendations [88]. Interestingly, adherence to guidelines in the Emergency Department was found lower if patients were women (70.5%) rather than men (73.4%), although adherence was similar in the subgroup of patients with pneumonia (63.5% in men vs 64.5% in women) [88]. It has been reported that the treating physician’s sex may influence the patient’s management: a study on 826 consecutive patients [89] found that female physicians admitted fewer patients to the ICU than male physicians (5% vs 10%) and female physicians’ patients received their first intravenous antibiotic dose later than male physicians’ patients. Nevertheless, the case fatality rate was the same in the two groups, and the authors suggest that this may show a more judicious patient assessment by female physicians. Not being linked to specific biological features, these differences can be considered gender-related, and deserve further attention and research in larger studies.

## COVID-19 pneumonia

Differences in incidence and outcomes between men and women have been reported since the first phases of the COVID-19 pandemic. Incidence was found higher in men from the beginning of the pandemic [90], but an early review (April 2020) [91] already showed that no sex differences could be found in the absolute number of COVID-19 cases. Nevertheless, in the subgroup of those older than 60 years, males were the most affected. Others [92] found similar results, with a female prevalence between the age of 10 and 50 years and a male prevalence before the age of 10 and after the age of 50. In the same study, the case fatality rate (CFR) was higher in males, with a male-to-female ratio of 3.1 in patients < 60 years old and 2.2 in those > 60. In July 2020, an analysis of data from 38 countries found that males’ CFR was 1.7 times higher than females [93], and the difference was persistent in all age groups > 30 years. Notably, a higher risk of death in men had been already reported in the previous coronavirus-caused pandemics, SARS [94], and MERS (Middle East Respiratory Syndrome) [95].

Apart from death, men were found to have a higher length of stay and higher rates of hospitalization, ICU admission, secondary bacterial infection, shock, vasopressor support, and endotracheal intubation [91, 96–98]. In both sexes, hypoxia was associated with increased mortality, while obesity and chronic kidney disease with an increased risk of intubation, but all these effects were larger in women [98]. Female patients were found more frequently with acute kidney injury and urinary tract infections [99].

These and other studies provided admirable insights, but most of the COVID-19 studies do not report sex-disaggregated data [100]. The most complete and up-to-date data source for sex difference in incidence and outcomes is the COVID-19 Sex-disaggregated Data Tracker [101] presented by the Global Health 50/50 Research Initiative. Using data on 180 countries (> 166 million patients, of which 23% were from the USA), completely available from this source, we estimated that 50.9% of affected patients were men on 1st December 2021; 74 countries shared sex-disaggregated data on hospitalized patients, which were men in 54.7% of cases [102]. Not surprisingly, ICU-admitted patients were mostly males in all countries [103].

As for the clinical severity of the disease, women are more frequently found with alteration of smell and taste [102] and prolonged symptoms (a condition known as “long-COVID”) [104], although their SARS-CoV-2 RNA shedding is shorter as compared with men [104]. On the other hand, men are more likely to present severe clinical manifestations, like neurological symptoms, venous thromboembolic events [105], or refractory disease [106]. Therefore, male sex has been included as a risk factor in several of the scores that have been developed for COVID-19 [107, 108].

However, the introduction of vaccines represents a new and still not fully explored variable in shaping COVID-19 differences among sexes. Women usually have a higher response to vaccines; however, a recent meta-analysis [109] found that vaccination was more effective in preventing COVID-19 disease in men than women. No significant difference was found in the placebo arm, suggesting a specific sex effect of the vaccines. Further research, however, is needed to confirm these findings, and should clarify whether this allegedly differential efficacy of vaccines modified sex distribution of incidence and poor outcomes [110].

Analogously to CAP, the difference between men and women can partially be explained by gender-related factors. Male sex tends to have lifestyle habits which increase risk (e.g., smoking [111], alcohol, and drugs abuse) and also a higher burden of comorbidities (especially T2DM, COPD, hypertension, cardiovascular diseases, and chronic kidney disease) [111]; men were also found to wash their hand less frequently than women [112] and, after retirement, spend more time in public places than women, who are, conversely, prone to living alone and experiencing social isolation [113].

Furthermore, men are more likely to downplay the potential risk of SARS-CoV-2, so they have implemented fewer behavioral changes than women [114], like avoiding at-risk contact with those outside home or wearing masks [115]. It has also been reported that wearing masks can be perceived as a sign of 'frailty’ or ‘weakness’ among some men [116]. This would sensibly be linked to a higher COVID-19 incidence in men, however, as we saw, was not found. Nevertheless, when a man is infected, he can easily transmit the virus to other family members [117], leveling out the possible incidence gap.

On the other hand, women more frequently have a caregiver role, both at home and within the health system, and may thus be over-exposed to SARS-CoV-2. Besides, they are probably more affected than men by the short- and long-term social effects of the pandemic, such as the higher domestic violence during quarantine or the limited working opportunities in the post-pandemic world [113]. Those gendered negative externalities should be considered by policymakers to prevent future repercussions of the pandemic.

However, even if gender does almost certainly play a role, the increased risk of death for men emerged cross-culturally among different countries, so biological determinants are probably more meaningful in shaping COVID-19 morbidity and mortality.

## Conclusion

In this review, we highlighted the current knowledge on sex and gender-specific differences in epidemiology, pathogenesis, and natural history of CAP. However, we also underlined the needs for further studies which could improve our knowledge on this topic.

Sex and gender could be considered as silent disease-modifiers, especially in infectious disease. Their role ranges from epidemiology to the outcomes, passing through etiology and prognostic scores. We have now limited knowledge to understand how these factors, mostly not modifiable, could intersect with the patient care, and how to include them in the risk assessment model to predict severity of CAP. Many steps still need to be taken and further studies are urgently needed to shed light on those aspects that are still unknown, to clarify the contribution of sex and gender in this clinical scenario.
